# TALBOT: A Track-Leg Transformable Robot

**DOI:** 10.3390/s22041470

**Published:** 2022-02-14

**Authors:** Wenzhi Guo, Jiandu Qiu, Xinrui Xu, Juan Wu

**Affiliations:** 1 College of Mechanical and Vehicle Engineering, Taiyuan University of Technology, Taiyuan 030024, China; guowz@connect.hku.hk (W.G.); qiu19834439698@163.com (J.Q.); 2 Department of Mechanical Engineering, Faculty of Engineering, The University of Hong Kong, Hong Kong, China; u3589555@connect.hku.hk

**Keywords:** track-wheel mechanism, central pattern generator (CPG), quadruped, transformable robot, Fast-Slam

## Abstract

This article introduces a tracked-leg transformable robot, TALBOT. The mechanical and electrical design, control method, and environment perception based on LiDAR are discussed. The original tracked-leg transformable structure allows the robot to switch between the tracked and legged mode to achieve all-terrain adaptation. In the tracked mode, TALBOT is controlled by the method of differential speed between the two tracked feet. In the legged mode, TALBOT is controlled based on a bionic control strategy of the central pattern generator to realize the generation and conversion of gait. In addition, the robot is equipped with a LiDAR, through sensor preprocessing and optimization of the slam mapping algorithm, so that the robot achieves a better mapping effect. We tested the robot’s motion performance and the slam mapping effect, including going straight and turning in tracked and legged modes and building a map in an indoor environment.

## 1. Introduction

Tracked and wheeled mobile methods have been widely used in ground mobile systems. Previous studies [1,2] have shown that tracked and wheeled movement methods have many advantages when driving on relatively flat terrain, such as a rapid and stable movement speed, a simple structure, and a control policy. As robots work in increasingly complex environments, the limitations of traditional tracked and wheeled vehicles have become more and more obvious. Their simple and robust design cannot provide enough versatility to adapt to many real-world terrains. On severely rugged terrain, the role of wheel and track will be severely lost, energy consumption will be greatly increased, and movement efficiency will be greatly reduced. Researchers modify the design of robots to increase passive or active degrees of freedom, making these robots more suitable for rugged terrain. The American NASA rover [3] has greatly increased the adaptability of the robot to the terrain through the innovative design of the bogie.

In order to overcome the shortcomings of tracked and wheeled robots, researchers have taken inspiration from nature and replaced track and wheel structures with leg structures. One method is to design a robot with an articulated leg structure that can maintain a stable state of motion when the robot is in a rugged environment. SIAVASH REZAZADEH et al. [4] selected biologically related templates in the natural world and proposed a general principle to design the leg structure to improve efficiency based on the template mechanism. S. Kim et al. [5] built a quadruped robot to realize the flexible movement. Jo ao Pedro Barreto et al. [6] proposed a simple method to solve the dynamic modeling problem of a six-leg robot. A. Saunders et al. [7] built a six-legged tree climbing robot, which realized the movement on the trunk. As early as 2008, the large dog [8] robot developed by Boston Dynamics realized quadruped robots’ fast and flexible movement. Another method is to design a leg with a simple general structure, such as the insect RHex series [9,10,11]. Tracked and wheeled robots generally need a contact surface when they are moving, while leg robots can rely on the fulcrum of their legs to complete the movement. It can achieve a better moving effect when facing the rugged and uneven ground. In addition, a four-legged robot has better comprehensive performance in the harsh environment. Due to the large number of feet, the kinematics and dynamics of the six-legged robot are more complex [12], so it is difficult to achieve good control accuracy. Moreover, due to the limited shape of the six-legged robot, it can only be made into small ones, so its application scenarios have many requirements, and the possibility of large-scale commercialization is not high. The two-legged robot requires high balance [13]. At present, various technologies are not mature. The four-legged robot has strong stability, it is flexible, and it could adapt to different terrain [14]. Its shape can be large or small, so its application scenarios are more extensive. However, the general legged robot has a complex structure, a slow motion speed, low efficiency, and higher requirements for motors, which limit the development of a legged robot [15].

By effectively combining the tracked and the legged motion mode, the terrain adaptability of the robot is increased while maintaining the higher efficiency. N Babu et al. [16] proposed a new type of tracked-leg hybrid robot, which increases the versatility of the robot by switching between the tracked and the legged mode. However, the robot is driven by two sets of motion structures, which are complicated in structure. Faliang Zhou et al. [17] proposed a hybrid mobile robot based on foldable wheels. When the robot encounters an obstacle, it folds the wheel into an oval shape to get over the obstacle. However, the diameter of the wheel is small, and when the size of the obstacle is large, it is difficult for the robot to achieve a good obstacle-crossing effect. Alan Mutka et al. [18] proposed a flipper-track robot. The robot is composed of four tracked arms and a fuselage. Rotating the tracked arms drives the robot to move. However, the degree of freedom of the robot’s motion structure is less. When encountering complex ground conditions, the robot is likely to roll over. Luca Bruzzone et al. [19] proposed a novel hybrid leg-wheel robot, which combines wheels, tracks, and legs to greatly expand the terrain applicability, but the robot structure is too complex, which greatly increases the maintenance cost. Xingguang Duan et al. [20] proposed a Small Wheel-Track-Leg Mobile Robot, which has good obstacle-crossing performance, but the overall control strategy is complicated, and obstacles need to be sensed in advance. Wei-Hsi et al. [21] proposed a TurboQuad Robot. The robot adopts a variable wheel structure. Compared with the folding of the robot wheels in [17], the TurboQuad robot splits and unfolds the wheels, which greatly increases the obstacle-crossing performance.

On the other hand, for TALBOT in the legged mode, the coordinated movement of each leg under different terrains is the key to the motion control. The central pattern generator is a biological neural circuit that produces rhythmic motion behavior in animals. The CPG control method does not require precise modeling of the walking environment, and the control system can generate stable rhythm signals without high-level signals and external feedback, which can mimic the walking method of animals very well. Longbai et al. [22] proposed a new cpg-based gait generation method for a hexapod bending robot, and this method can achieve smooth gait transitions between multi-mode gaits; Haitao Yu et al. [23] also achieved a similar effect. A. Crespi et al. [24] used a CPG based on limit cycles to process signal and generate rhythmic trajectories, which were applied to Salamandra Robotica. Many quadruped robot control strategies using cpg have been studied. For example, Yinquan Zeng et al. [25] proposed a quadruped robot bionic control strategy based on the Central Pattern Generator—Neural Network Workspace Trajectory (CPG-NN-WT). Chengju liu et al. [26] generated an adaptive walking pattern based on a multi-layer cpg network to achieve a good control effect. Takahiro Fukui et al. [27] combined vestibular feedback and central pattern generators (CPG) to realize the speed-based autonomous gait transition and autonomous and stable running of the quadruped robot. In addition, cpg has also been applied to snake-shaped robots [28,29,30], fish-shaped robots [31,32], and biped robots [33,34].

**Figure 1:**
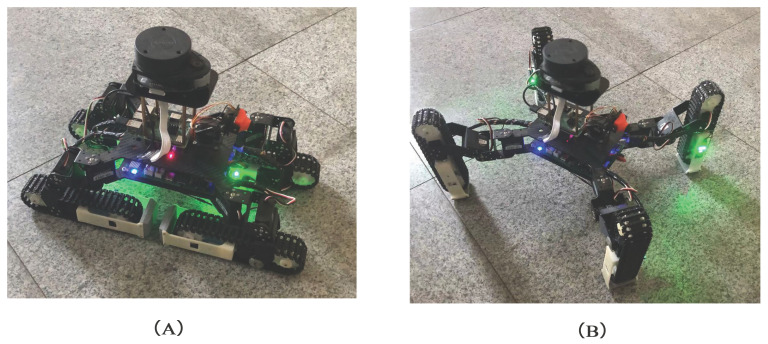
(**A**) The tracked mode of TALBOT. (**B**) The legged mode of TALBOT.

This article introduces a hybrid tracked-leg robot, TALBOT. Figure 1A,B shows TALBOT in the tracked mode and the legged mode. We designed a new type of track-leg conversion mechanism. The tracked foot structure can act as a part of the leg in the legged mode and drive the tracks in the tracked mode, which simplifies the movement structure. In addition to the new mechanism, we propose a complete control strategy for two motion modes. When the robot is in the tracked mode, the robot is controlled by a tracked foot differential on both sides. When the robot is in the legged mode, the hopf oscillator is selected as an oscillation unit of the CPG network, and the CPG control scheme is designed and improved to control a variety of motion gaits of TALBOT. The network coordinates the four legs to achieve a smooth and stable control effect. In addition, TALBOT is also equipped with a LiDAR. We used the least square method to fuse the odometer of the wheel and the Inertial Measurement Unit (IMU) to preprocess the sensor data, which obtain more accurate position information than a single wheel odometer and reduce the non-systematic error. Then, the proposal distribution was optimized based on a particle filter algorithm. LiDAR matching was used to represent the proposal distribution, so that the sampling range was limited to a relatively small range, and the effect of mapping was improved.

The composition of this article is as follows. The second section mainly introduces the hardware composition of TALBOT, which focuses on the innovative design and conversion principle of the track-leg structure. The third section mainly introduces the software design, including the control strategy of the robot in tracked and legged modes and the improved Fast-Slam algorithm based on the particle filter. The fourth section mainly introduces the experimental evaluation results, including the moving straight and turning experiment, and the effect of building a map using the optimized Fast-Slam algorithm. The fifth section summarizes the full text.

## 2. TALBOT Hardware Design

We introduce the novel mechanical design of the track-leg robot: (i) Tracked foot structure and the transformation mechanism. (ii) Infrastructure of TALBOT.

### 2.1. Tracked Foot Structure and the Transformation Mechanism

**Figure 2:**
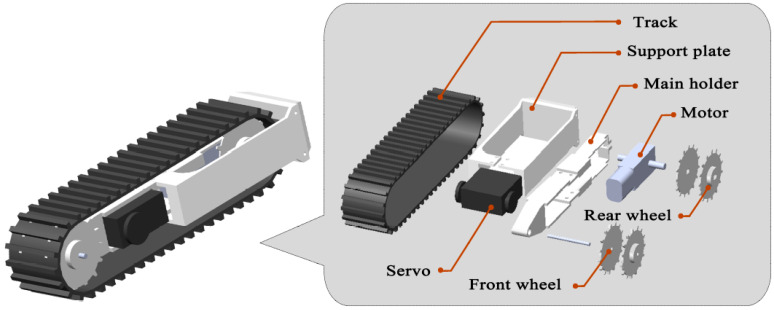
The tracked foot structure.

The structure of TALBOT is symmetrical from left to right. It consists of four tracked legs, and each tracked leg is composed of three servos and a tracked foot structure. The tracked foot is composed of four wheels, as shown in Figure 2. The two wheels at the back are driven by motors, and the other wheels at the front follow the track to rotate. The motor is equipped with a small deceleration device, which increases the torque of the motor and gives TALBOT more power. The position of the servo is shown in Figure 2, and the servo is rigidly connected to the tracked foot. The motion of the tracked foot can be controlled by rotating the servo. The outermost part of the tracked foot is the support plate. This component directly supports the movement of the robot and is subject to friction in contact with the ground. A certain degree of rigidity, strength, and wear resistance must be guaranteed to avoid deformation or even breakage during the movement, which reduces the useful life of TALBOT.

**Figure 3:**
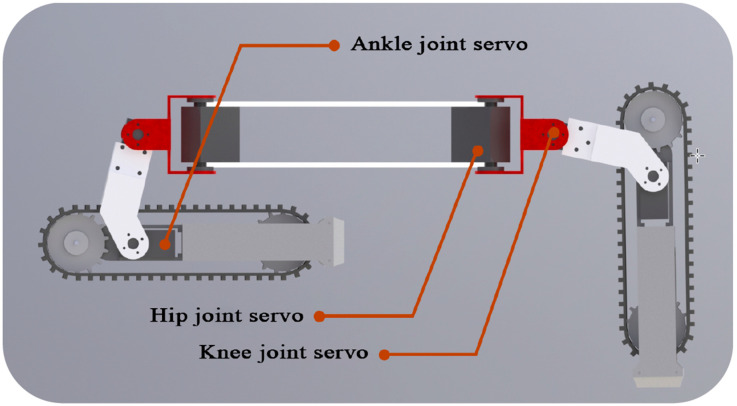
The front view of the leg structure.

The four legs are connected by upper and lower plates of the same shape, and the two plates mainly play a connection and supporting role. Each tracked leg can be driven individually or coordinated. Stripping off the sensor and controller, the front view of the leg structure is shown in Figure 3. The rotation axis of the hip and knee joint servo are perpendicular to each other; the rotation axis of the knee and ankle joint servo are coincident; and the U-shaped connector is selected for connection. By controlling the rotation angle of the servo and the rotation of the motor, the four track legs move coordinately to complete the expansion, forward, backward, steering, retraction, obstacle crossing, and other movements. Table 1 shows the rotation angle of each servo.

The unfolding and folding movement of the tracked legs is the dividing line between the transition of the legged and the tracked mode. The unfolding movement of the four tracked legs makes TALBOT switch from the tracked mode to the legged mode. The hip joint servo is controlled to rotate to the unfolding angle, and then the ankle and knee joint servos of the track legs are simultaneously controlled to rotate to make the tracked foot expanded at a certain angle relative to the body to achieve landing support.

**Figure 4:**
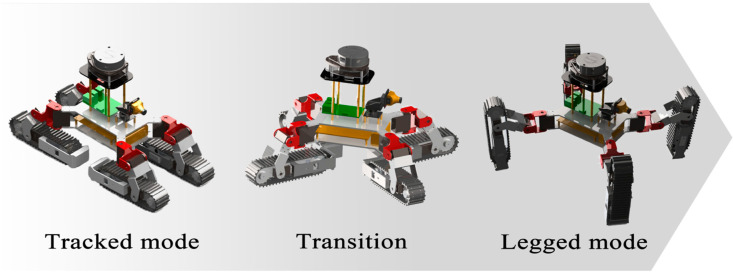
The transition process.

The folding motion of the tracked legs makes TALBOT switch from the legged mode to the tracked mode. The robot moves on four legs before retraction. When the motion is stopped, the position of each leg cannot meet the requirements of direct retraction. First, reset the tracked foot to their initial position after deployment and then retract them. The transition from the tracked mode to the legged mode is shown in Figure 4.

### 2.2. Infrastructure of TALBOT

In order to measure the movement of TALBOT, the motor in each tracked foot is equipped with a high-precision rotary encoder to implement a wheel encoder odometer. The odometer gives the position of TALBOT (*x*, *y*, δ), where (*x*, *y*) represents the position; δ represents the position in the global coordinate system; and the movement state is (*v*, ω), where *v* is the linear velocity, and ω is the angular velocity.

TALBOT is equipped with LiDAR to sense the surrounding environment, which has the characteristics of a wide detection range and a high recognition rate. The LiDAR is installed above the camera to ensure that the LiDAR is not blocked during scanning.

The robotic electromechanical system consists of several components. As the main processor, the Raspberry Pi is responsible for receiving motor speed information, converting motor speed information to the odometer information through certain relationship, steering gear rotation information, collecting LiDAR data, and processing LiDAR information through inter frame matching and other methods. STM32 receives the motor speed information and steering gear angle information from Raspberry Pi, sends it to the motor and the steering gear controller, and drives the actuator to perform corresponding operations. In addition, the STM32 MCU uses the 433 wireless communication protocol to connect to the remote control, which can directly control the movement of the robot. Table 2 shows the specifications of robot.

## 3. TALBOT Software Design

We propose the detailed design of the intelligent control software of TALBOT: (i) movement control. (ii) The optimization of Fast-Slam.

### 3.1. Movement Control

#### 3.1.1. Tracked Mode Movement Control

Because the four tracked legs are directional, TALBOT can turn around by changing the direction of the tracked leg feet. However, this method has too much load on the servos, so the robot’s movement control is carried out by the method of differential speed on both sides of the tracks. When the speeds of the tracks on both sides are different, TALBOT turns to the slower side. Using this method, the turning radius can be 0, which achieves the effect of turning in place. The speed of the tracked feet on the left and right sides is $v_{r}$ and $v_{l}$, respectively, and the overall speed of TALBOT is

(1) $$v=\frac{v_{R}+v_{L}}{2}$$

TALBOT has four driving tracked feet; the diameter of the wheel in the tracked foot is *d*; the photoelectric encoder is *p* line/revolution; the pulse frequencies output by the photoelectric encoders of the left and right driving wheels are $f_{L}$ and $f_{R}$, respectively; and the linear velocity of the wheel is

(2) $$\begin{array}{c} v_{R}=\frac{f_{R}}{p}\times \pi \times d,\;v_{L}=\frac{f_{L}}{p}\times \pi \times d \end{array}$$

The distance between the left and right tracked feet is *L*, and the turning radius of the left and right tracked feet of TALBOT are $R_{L}$ and $R_{R}$, respectively; So

(3) $$\begin{array}{c} R_{R}=R_{L}+L \end{array}$$

The angular velocity is

(4) $$\begin{array}{c} \begin{array}{c} w=\frac{v_{R}}{R_{R}}=\frac{v_{L}}{R_{L}}=\frac{v_{R}-v}{L} \end{array} \end{array}$$

If the robot moves from state $X_{n}={\left(x_{n},y_{n},\varphi_{n}\right)}^{T}$ to state $X_{n+1}={\left(x_{n+1},y_{n+1},\varphi_{n+1}\right)}^{T}$, the angle variation value $\Delta \varphi_{n}$, the arc length $l_{n}$ through which the robot moves in circular motion, and the corresponding chord length $s_{n}$ can be obtained, respectively:

(5) $$\begin{array}{c} \begin{array}{c} \Delta \varphi_{n}=w_{n}\Delta t\\ l_{n}=w_{n}\Delta t_{n}\left(R_{L}+\frac{L}{2}\right)\\ s_{n}=\frac{L\left(v_{R_{n}}+v_{L_{n}}\right)}{2\left(v_{R_{n}}-v_{L_{n}}\right)}\sin\frac{\Delta \varphi_{n}}{2} \end{array} \end{array}$$

From this, the state change from *n* to *n* + 1 is

(6) $$\begin{array}{c} \begin{array}{c} \Delta x_{n}=\frac{L\left(v_{R_{n}}+v_{L_{n}}\right)}{2\left(v_{R_{n}}-v_{L_{n}}\right)}\sin\frac{\Delta \varphi_{n}}{2}\cos\left(\varphi_{n}+\frac{\Delta \varphi_{n}}{2}\right)\\ \Delta y_{n}=\frac{L\left(v_{R_{n}}+v_{L_{n}}\right)}{2\left(v_{R_{n}}-v_{L_{n}}\right)}\sin\frac{\Delta \varphi_{n}}{2}\sin\left(\varphi_{n}+\frac{\Delta \varphi_{n}}{2}\right) \end{array} \end{array}$$

The equation of motion is

(7) $$\begin{array}{c} X_{n+1}=\left[\begin{array}{c} x_{n+1}\\ y_{n+1}\\ \varphi_{n+1} \end{array}\right]=\left[\begin{array}{c} x_{n}+\Delta x_{n}\\ y_{n}+\Delta y_{n}\\ \varphi_{n}+\Delta \varphi_{n} \end{array}\right]\\ =\left[\begin{array}{c} \frac{L\left(v_{R_{n}}+v_{L_{n}}\right)}{2\left(v_{R_{n}}-v_{L_{n}}\right)}\left(\sin\varphi_{n+1}-\sin\varphi_{n}\right)\\ \frac{L\left(v_{R_{n}}+v_{L_{n}}\right)}{2\left(v_{R_{n}}-v_{L_{n}}\right)}\left(\cos\varphi_{n+1}-\cos\varphi_{n}\right)\\ \varphi_{n}+\frac{v_{R_{n}}-v_{L_{n}}}{L}\Delta \end{array}\right] \end{array}$$

#### 3.1.2. Legged Mode Movement Control

Gait refers to the movement of a foot-type robot in which one leg lifts and falls in accordance with certain rules in coordination to realize the body displacement in space. The gait plays a vital role in the movement of the legged robot. During the movement of TALBOT in the legged mode, the leg includes two movement states: the stance phase and the swing phase. The swing phase refers to the process in which the leg moves forward to complete a forward motion, while the support phase is the process in which the leg remains stationary to maintain the stability.

When the traveling speed is slow, each step of the quadruped animal is in a relatively stable three-legged support state, such as a tortoise. This movement pattern is called walk gait. The faster ones are the trot and pace gaits. In order to ensure the smooth operation of TALBOT, only the slower walk and trot gaits were studied.

The defined gait period *T* is the time required for all legs of TALBOT to act as a swinging leg, that is, the time required for a swinging leg to act as swinging leg again. The ratio of the time a leg is used as a supporting leg in a walking cycle to the cycle time *T* is defined as the support factor β, as shown in equation:

(8) $$\beta =\frac{t_{1}}{T}=1-\frac{t_{2}}{T}$$

where $t_{1}$ is the duration of the stance leg in a period *T*. $t_{2}$ is the duration of the swing leg in a period *T*. *T* = $t_{1}$ + $t_{2}$ is the time of one gait cycle of TALBOT.

It can be seen from Formula (8) that the value of β is between 0 and 1. According to the value of β, the gait of TALBOT is also different.

(1) When $0\leq \beta <\frac{1}{2}$, the number of stance legs of the TALBOT was always less than 2, and sometimes the number of stance legs was 0, which was an unstable gait. Although the speed of TALBOT under this gait was the fastest, the stability was the worst
(2) When β = $\frac{1}{2}$, the time of the swing phase is equal to the time of the stance phase at this time. During the whole movement, TALBOT always keeps two legs in a stance state to maintain its own stable state, and the other two legs in a swing state can maintain its own forward speed. This gait is called a trot or pace gait.
(3) When $\frac{1}{2}<\beta <\frac{3}{4}$, TALBOT is in the transition state two-legged gait and three-legged gait.
(4) When β = $\frac{3}{4}$, TALBOT will have three legs in the stance state and the other leg in the swinging state. Compared with the two-leg gait, the number of supporting legs in this state is more, so it is more stable in the process of walking, but the forward speed in the process of movement will be slower. This state is called walk gait.
(5) When $\frac{3}{4}<\beta <1$, three legs of TALBOT are in the stance state, and the other leg is in the transition state between the swing state and the stance state.
(6) When β = 1, all four legs of TALBOT are in a stance state, that is, a static state

TALBOT uses the hopf oscillator as the core to establish the motion control model of the CPG. The hopf oscillator is a harmonic oscillator with fewer parameters and a clear physical meaning. Each parameter individually affects the performance of the oscillator and is convenient for tuning. The amplitude, frequency and phase of the output signal are easy to control and can be used to control the walking gait. Its mathematical expression is

(9) $$\left\{\begin{array}{c} \dot{x}=\alpha \left(\mu -x^{2}-y^{2}\right)x-\omega y\\ \dot{y}=\alpha \left(\mu -x^{2}-y^{2}\right)y-\omega x \end{array}\right.$$

where *x* and *y* are the state variables of the oscillator, that is, the output of the oscillator. α is the convergence rate coefficient, which is used to control the convergence rate of the limit cycle and is a normal number. The larger α is, the faster the limit cycle can converge. μ is the square of oscillator amplitude; ω is the frequency of the oscillator

(10) $$\left\{\begin{array}{c} \omega =\frac{\omega_{st}}{e^{(}-ay)+1}+\frac{\omega_{sw}}{e^{(}ay)+1}\\ \omega_{st}=\frac{1-\beta }{\beta }\omega_{sw} \end{array}\right.$$

**Figure 5:**
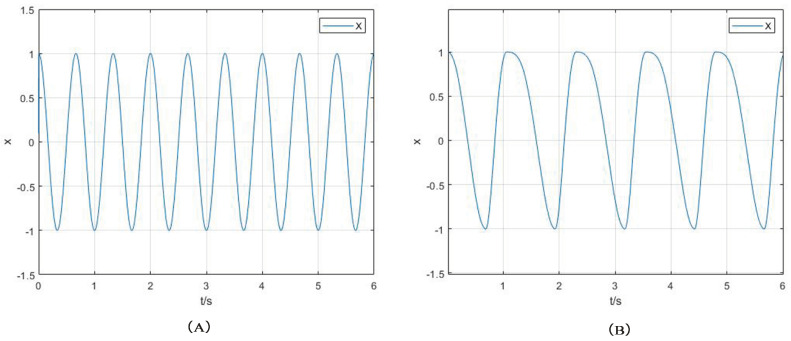
(**A**) shows the the x output, when β = $\frac{1}{2}$. (**B**) shows the the x output, when β = $\frac{3}{4}$.

Among them: $\omega_{st}$ is the stance phase frequency; $\omega_{sw}$ is the swing phase frequency; *a* is a larger normal number, which determines the conversion speed of $\omega_{st}$ between $\omega_{st}$ and $\omega_{sw}$; and β is the support factor. When β = $\frac{1}{2}$, the swing time is the same as the stance time. By changing the value of β, the swing time and the stance time can be adjusted. Taking β = $\frac{1}{2}$, *a* = 100, $\omega_{sw}$ = 3π, μ = 1, the output curve of the oscillator state variable *x* is shown in Figure 5A. Taking β = $\frac{3}{4}$, it can be seen from Figure 5B that the swing time is different from the stance time.

**Figure 6:**
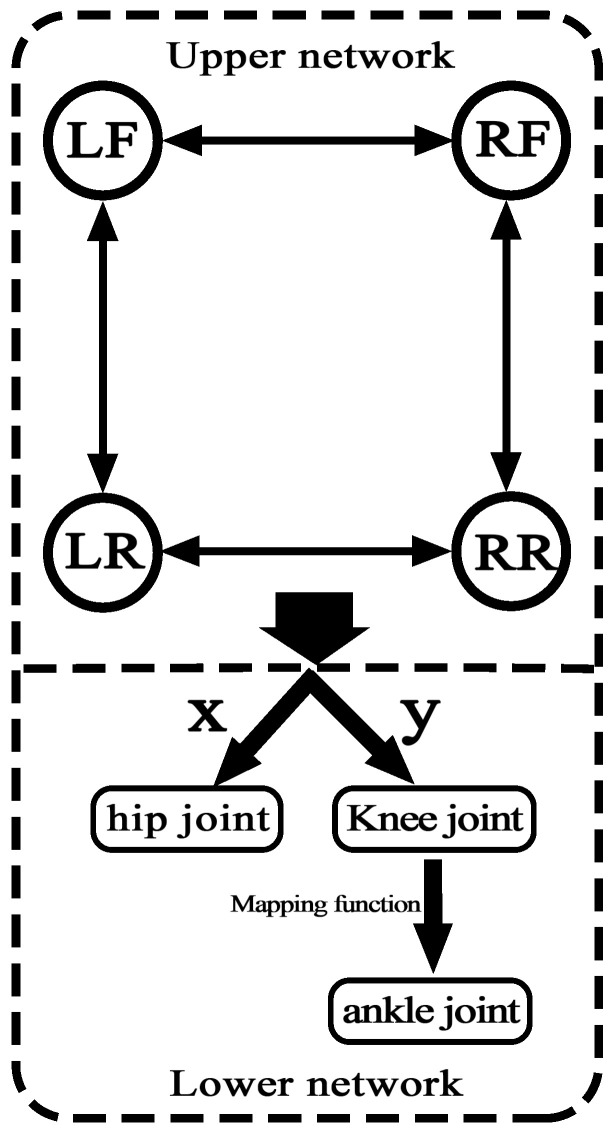
The hierarchical control network of CPG.

To realize the coordinated movement between the legs, it is necessary to construct a CPG network, through the mutual coupling of multiple oscillators, to ensure the synchronization and coordination of the movement. Generally, a common method to generate gait is to use 12 oscillators to form a fully symmetrical CPG network. Each oscillator corresponds to a robot joint, and the joints are coupled to each other to form a phase difference. So, the CPG network is complicated, and parameter adjustment is difficult. Aiming at the characteristics of the quadruped TALBOT’s gait, the CPG coupling model in this study adopts a hierarchical model. As shown in Figure 6, the upper layer of the CPG network is the coupling between the legs, and the network connection mode is a ring network connection, which only requires four oscillators. The bottom layer is the CPG intra-leg coupling, which is composed of hip joints and corresponding knee joints. The ankle joint control signal is realized by the knee-ankle mapping function.

Let the rotation angles of the hip, knee, and ankle joints be $\theta_{1}$, $\theta_{2}$, and $\theta_{3}$, respectively. The rotation ranges of the hip, knee, and ankle joints are as shown in the Table 1, and the mapping function between them and the output curve of the oscillator is

(11) $$\left\{\begin{array}{c} \theta_{1}=k_{0}x\\ \theta_{2}=\left\{\begin{array}{cc} k_{1}y+b_{1} & y⩾0\\ k_{2}y+b_{2} & y<0 \end{array}\right.\\ \theta_{3}=k_{3}\theta_{2}+b_{3} \end{array}\right.$$

**Figure 7:**
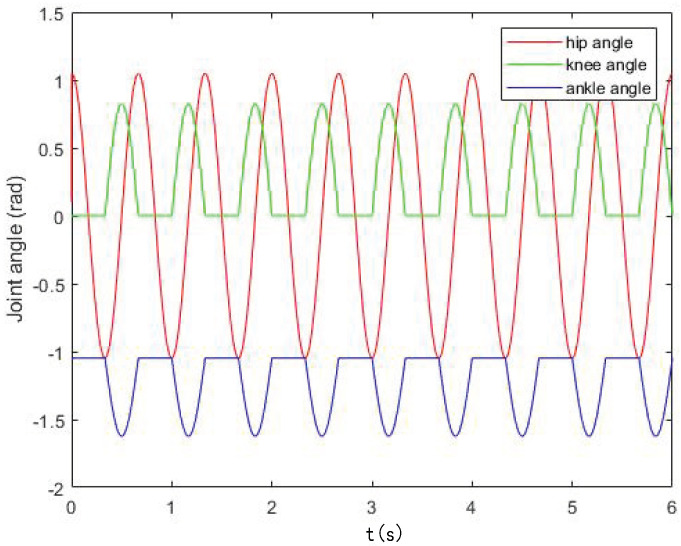
The joint angles of robot.

Among them: $k_{0}$ is the mapping coefficient of the hip joint; $k_{1}$ and $k_{2}$ are the mapping coefficients of the knee joint; and $k_{3}$ is the mapping coefficient of the ankle joint, which is used to adjust the amplitude of the joint control signal. Combining the mechanical structure and parameters of the robot in this study, take β = 0.5, $k_{0}$ = $\frac{\pi }{3}$, $k_{1}$ = $\frac{\pi }{4}$, $b_{1}$ = 0, $k_{2}$ = 0, $b_{2}$ = 0, $k_{3}$ = 0.7, and $b_{3}$ = −$\frac{\pi }{3}$ and obtain the joint angle control signals of each joint as shown in Figure 7.

The oscillators that control the four legs of the TALBOT are coupled with each other and continuously output joint rotation control signals, so that TALBOT can move in various gaits. This section uses the ring-type coupling network topology to describe the phase coupling relationship between the output signals of each oscillator model. Combining Formulas (9) and (10), its mathematical model is

(12) $$\left\{\begin{array}{c} {\dot{x}}_{i}=\alpha \left(\mu -x_{i}^{2}-y_{i}^{2}\right)x_{i}-\omega_{i}y_{i}\\ {\dot{y}}_{i}=\alpha \left(\mu -x_{i}^{2}-y_{i}^{2}\right)y_{i}+\omega_{i}x_{i}+\lambda \left(y_{j}\cos\theta_{j}-x_{j}\sin\theta_{j}\right)\\ \omega_{i}=\omega_{\mathrm{st}\;}/\left(e^{-\alpha y_{i}}+1\right)+\omega_{\mathrm{sw}\;}/\left(e^{\alpha_{yi}}+1\right) \end{array}\right.$$

Among them: λ is the coupling strength parameter between the two oscillators. The value of λ will affect the connection between the rising and falling sections of the output curve. In order to prevent glitches in the output curve and cause chattering in the system, the value of λ should not be too high. TALBOT takes λ = 0.6; $\theta_{ji}$ is the phase difference between oscillator *i* and *j*, that is, $\theta_{ji}$ = $\theta_{i}$−$\theta_{j}$; the definition of other parameters is consistent with Formulas (9) and (10).

**Figure 8:**
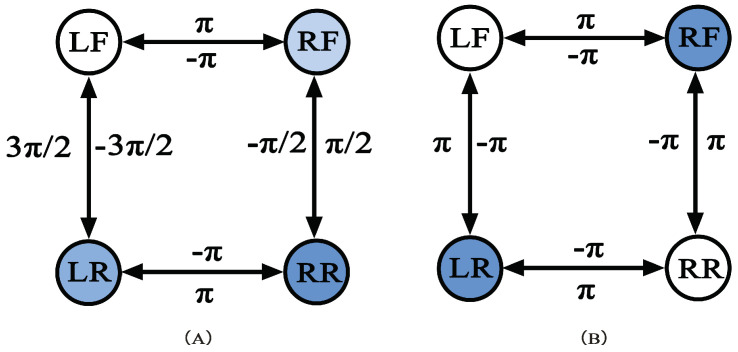
The control network of CPG in walk (**A**) and trot gait (**B**).

When TALBOT walks in a trot gait, its legs are divided into legs LF, RR, and legs RF, LR. The two legs of the same group have the same phase, and the two legs that are not in the same group have a phase difference of π. The coupling network is shown in Figure 8B; when walking in a walk gait, each leg of the robot is a group; enters the swing phase in the order of LF, RF, LR, RR; and the phase difference between the two legs of the adjacent group is $\frac{\pi }{2}$; its ring-shaped coupling network is as shown in Figure 8A.

**Figure 9:**
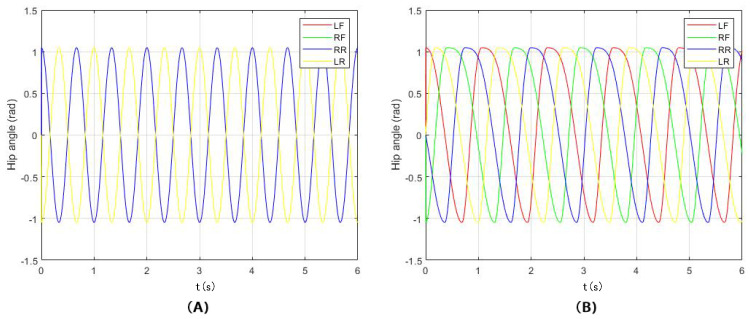
(**A**) shows the CPG output in walk gait. (**B**) shows the CPG output in tort gait.

Set the stance factor β = 0.5 and 0.75 for the walk and trot gaits, respectively, and set the other parameters according to the relevant parameters of TALBOT and the output curve of the hip joint oscillator as shown in the Figure 9A,B, respectively.

**Figure 10:**
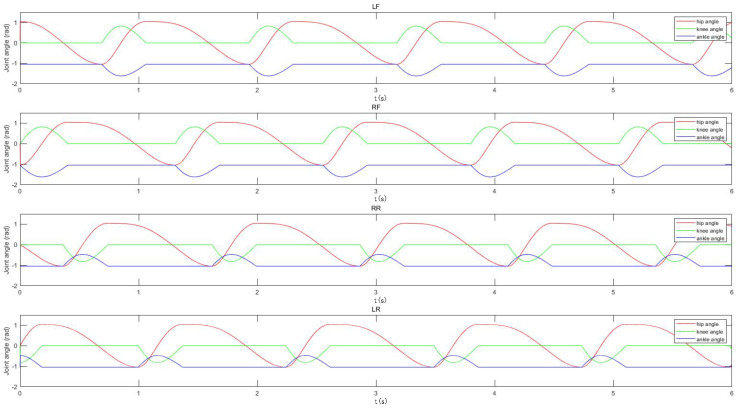
The joint angles in walk gait.

**Figure 11:**
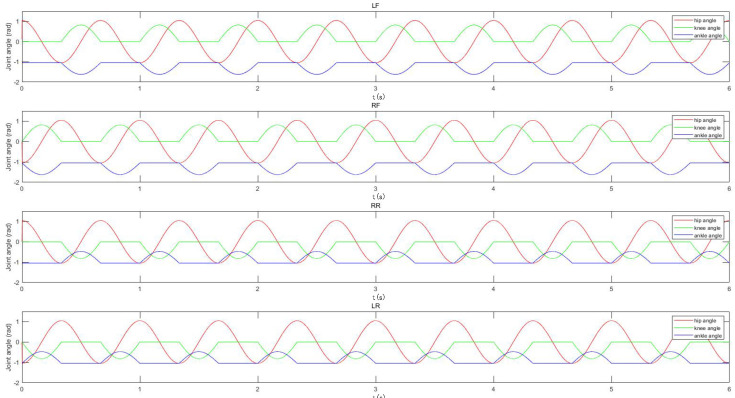
The joint angles in tort gait.

According to Formula (11), the angles of the ankle joint are obtained through the mapping of the knee joint, so as to obtain the rotation angle of each joint of the robot in the walk and trot gait, as shown in Figure 10 and Figure 11. In addition, by adjusting the amplitude and the positive or the negative of the output curve of the oscillator, TALBOT can turn around and back up.

### 3.2. The Optimization of Fast-Slam

When TALBOT is in the legged mode, the LiDAR is difficult to maintain stability as the robot shakes, and the odometer is difficult to estimate accurately, so the mapping effect is not good. Therefore, this study only researched the optimized Fast-Slam algorithm to build a map when TALBOT is in the tracked mode. We used the following two methods to improve the Fast-Slam building map effect: (i) a data preprocess and (ii) an improved particle filter positioning algorithm.

#### 3.2.1. Data Preprocess

The accuracy of odometer data directly affects the effect of building a map.The process of calculating a wheeled odometer usually does not require environmental information, and its accuracy is mainly limited by the accuracy of the sensor itself. Of course, the observation accuracy will decrease in uneven and slippery places. Since the LiDAR data are an observation of the external environment, although the LiDAR measurement accuracy is very high, it is extremely vulnerable to environmental influences, such as long corridors that cannot infer the movement in the direction of the corridor. In order to compensate for the error of the odometer, the odometer data were subjected to the least linear squares to eliminate the system error. $u_{i}^{*}$ is the data estimated by the LiDAR scan-match, and $u_{i}$ is the movement information of TALBOT measured by the odometer. Assuming a linear relationship between them, we can get

(13) $$u_{i}^{*}=X^{*}u_{i}$$

For each set of data, we can get the matrix

(14) $$\left[\begin{array}{ccccccccc} u_{ix} & u_{iy} & u_{i\varphi } & 0 & 0 & 0 & 0 & 0 & 0\\ 0 & 0 & 0 & u_{ix} & u_{iy} & u_{i\varphi } & 0 & 0 & 0\\ 0 & 0 & 0 & 0 & 0 & 0 & u_{ix} & u_{iy} & u_{i\varphi } \end{array}\right]\left[\begin{array}{c} x_{11}\\ \vdots \\ x_{33} \end{array}\right]=\left[\begin{array}{c} u_{ix}{}^{*}\\ u_{iy}{}^{*}\\ u_{i\varphi }{}^{*} \end{array}\right]$$

(15) $$\begin{array}{cc} \; & A=\left[\begin{array}{ccccccccc} u_{ix} & u_{iy} & u_{i\varphi } & 0 & 0 & 0 & 0 & 0 & 0\\ 0 & 0 & 0 & u_{ix} & u_{iy} & u_{i\varphi } & 0 & 0 & 0\\ 0 & 0 & 0 & 0 & 0 & 0 & u_{ix} & u_{iy} & u_{i\varphi } \end{array}\right]\\ \; & B=\left[\begin{array}{c} u_{\dot{x}x}{}^{*}\\ u_{i\bar{y}y}{}^{*}\\ u_{i\varphi } \end{array}\right] \end{array}$$

The correction matrix is

(16) $$X={\left(A^{T}A\right)}^{-1}A^{T}B$$

When TALBOT is moving, wheel slip or bumps will cause the encoder data to be distorted, which will affect the wheel odometer data and ultimately lead to the unsatisfactory effect of the robot’s mapping. In order to overcome these conditions, the Extended Kalman Filter (EKF) algorithm was used to integrate the wheel odometer data with the IMU.

**Figure 12:**
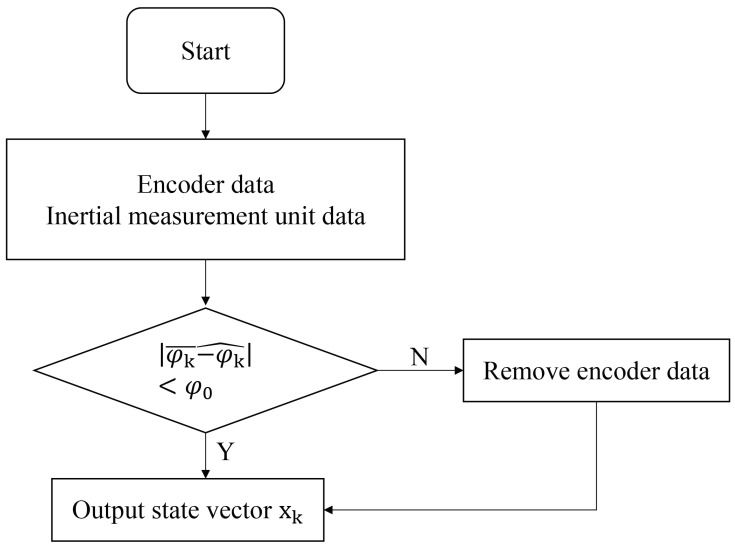
The EKF data flow.

We input the encoder data (translational linear velocity, rotational angular velocity, and yaw angle) and inertial measurement unit data (translational linear velocity, rotational angular velocity, and yaw angle) after simple data processing by setting the yaw angle threshold to determine whether TALBOT is slipping or bumping. When the difference between the yaw angle and the yaw angle is greater than or equal to the set threshold, it means that TALBOT has slipped or bumped; then, the input data of the encoder at the moment are removed; only the data of the inertial measurement unit are used; and the state vector is output through the EKF algorithm. When the difference between the yaw angle calculated by the encoder and the yaw angle calculated by the inertial measurement unit is less than the set threshold, the EKF algorithm is used to fuse the encoder data and the inertial measurement unit data and output the state vector. The flow of data is shown in Figure 12.

#### 3.2.2. Improved Particle Filter Positioning Algorithm

The Fast-Slam algorithm estimates the position of the robot through particle filtering and then calculates a map for each particle separately. Therefore, each particle contains the robot’s trajectory $x_{1:t}$ and the corresponding environment map. Converting its estimation of $x_{1:t}$ into an incremental estimation problem, the algorithm flow is:

(17) $$\begin{array}{cc} P\left(x_{1:t}|u_{1:t},z_{1:t}\right) & =\eta p\left(z_{t}|x_{1:t},z_{1:t-1},u_{1:t}\right)p\left(x_{1:t}|z_{1:t-1},u_{1:t}\right)\\ \; & =\eta p\left(z_{t}|x_{t}\right)p\left(x_{1:t}|z_{1:t-1},u_{1:t}\right)\\ \; & =\eta p\left(z_{t}|x_{t}\right)p\left(x_{t}|x_{1:t-1},z_{1:t-1},u_{1:t}\right)p\left(x_{1:t-1}|z_{1:t-1},u_{1:t}\right)\\ \; & =\eta p\left(z_{t}|x_{t}\right)p\left(x_{t}|x_{t-1},u_{t}\right)p\left(x_{1:t-1}|z_{1:t-1},u_{1:t-1}\right) \end{array}$$

In the formula, $p\left(x_{1:t-1}|{z_{1:t-1},u}_{1:t-1}\right)$ is represented by a particle swarm, and each particle is propagated by kinematics model $p\left(x_{t}|{x_{t-1},u}_{t}\right)$. For the propagated particles, the observation model is used to calculate the weight, and the map is constructed according to the estimated pose.

The mapping algorithm is currently the most widely used 2D LiDAR SLAM algorithm, which can achieve better mapping effects in a smaller environment. However, the Gmapping algorithm only optimizes the particle dissipation problem and dimensionality disaster of the particle filter based on the Fast-Slam algorithm. Therefore, based on the Gmapping algorithm, the proposal distribution was researched and optimized to improve the robot’s positioning and mapping accuracy.

**Figure 13:**
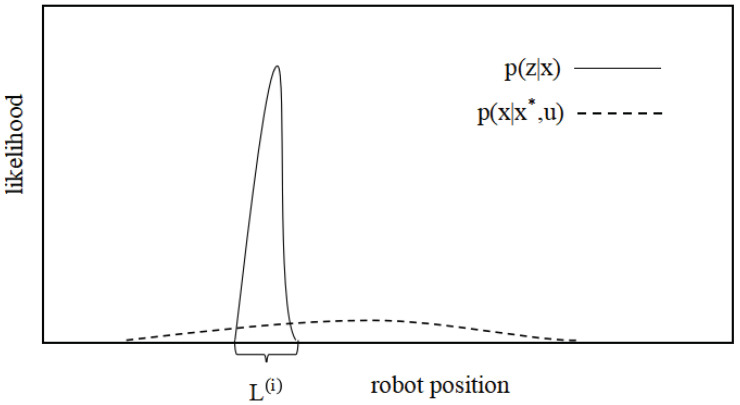
Proposal distribution.

The matching of LiDAR is much more accurate than that of the odometer. In terms of distribution, the variance of LiDAR matching is much smaller than that of the odometer model. If the proposal distribution is represented by LiDAR matching, the sampling range can be limited to a relatively small range, and the probability distribution of the robot can be covered with fewer particles, as shown in Figure 13.

The robot’s proposal distribution pose probability at time *t* is

(18) $$\begin{array}{cc} p\left(x_{t}|x_{t-1},u_{t},z_{t},m\right)= & \eta p\left(z_{t}|x_{t},m\right)p\left(x_{t}|x_{t-1},u_{t}\right) \end{array}$$

In the formula, $p\left(z_{t}|x_{t},m\right)$ is dominant in its own area $\left(L^{\left(i\right)}\right)$, and at this time $p\left(x_{t}|{x_{t-1},u}_{t}\right)$ is no longer important. Let it be a constant, so:

(19) $$\begin{array}{cc} p\left(x_{t}|x_{t-1},u_{t},z_{t},m\right) & =\eta p\left(z_{t}|x_{t},m\right)x_{t}\in L^{\left(i\right)} \end{array}$$

We changed the proposal distribution from the odometer observation model to the LiDAR observation model. The variance in the LiDAR observation model was small. Assuming that it obeys the Gaussian distribution, scan-match ($p\left(z_{t}|x_{t},m\right)$) and maximum likelihood estimation were combined to obtain the local extremum, considering $x_{t}^{*}$ is relatively close to the mean of the Gaussian distribution and sampling around $x_{t}^{*}$ to get *k* poses; it was considered that all *k* poses are in the Gaussian distribution:

(20) $$\left\{x_{j}∥x_{j}-x_{t}^{*}|<\Delta \right\}$$

In the formula, $x_{j}$ is the estimated pose of the robot under the *j*-th particle, and δ is a tiny amount. Score these *k* poses $p\left(z_{t}|x_{j},m\right)$, and consider these *k*. The pose obeys the Gaussian distribution, and the Gaussian distribution expression can be obtained as

(21) $$\begin{array}{c} \mu =\frac{1}{n}\sum_{j=1}^{k}x_{j}p\left(z_{t}|x_{j},m\right)\\ \Sigma =\frac{1}{n}\sum_{j=1}^{k}\left(x_{j}-u\right){\left(x_{j}-u\right)}^{T}p\left(z_{t}|x_{j},m\right) \end{array}$$

The proposal distribution becomes the Gaussian distribution *N*(μ,Σ), so the particle propagation is modified from the kinematic model sampling to the Gaussian distribution sampling.

(22) $$\begin{array}{c} w=\eta \frac{p\left(z_{t}|x_{t},m\right)p\left(x_{t}|u_{t},x_{t-1}^{i}\right)\mathrm{bel}\left(x_{t-1}\right)}{p\left(x_{t}|x_{t-1},u_{t},z_{t},m\right)bel\left(x_{t-1}\right)}\\ p\left(x_{t}|x_{t-1},u_{t},z_{t},m\right)=\frac{p\left(z_{t}|x_{t},m\right)p\left(x_{t}|u_{t},x_{t-1}\right)}{p\left(z_{t}|x_{t-1},u_{t},m\right)} \end{array}$$

Therefore, the weight calculation can be simplified as:

(23) $$w=p\left(z_{t}|x_{t-1},u_{t},m\right)=\sum_{j=1}^{j=k}p\left(z_{t}|x_{t},m\right)$$

## 4. Experiment

In this section, we conduct experiments for motion control and map building functions of TALBOT.

### 4.1. Moving Straight

**Figure 14:**
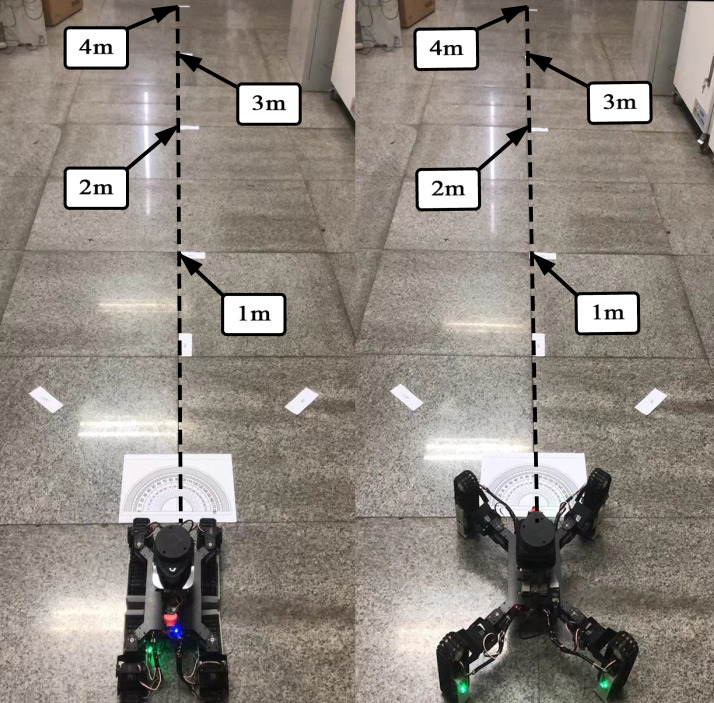
The moving straight experiment in tracked and legged modes.

In order to test the control effect of the robot, we conducted a moving straight experiment in the tracked mode and the legged mode and recorded the actual pose. The moving straight experiment is shown in Figure 14.

Due to the processing, installation, and size measurement, there were systematic errors, so the robot in the actual movement cannot achieve complete accuracy. The size and the angle of the deviation from the straight line when driving 1 m, 2 m, 3 m, and 4 m distances are shown in Table 3 and Table 4.

The error $E_{t}$ $E_{l}$ in tracked and legged modes calculation of the robot is as follows:

(24) $$\begin{array}{cc} E_{t}=\bar{E_{t}} & =\frac{\frac{\alpha_{11}}{1}+\frac{\alpha_{12}}{2}+\frac{\alpha_{13}}{3}+\frac{\alpha_{14}}{4}+\frac{\alpha_{21}}{1}+\frac{\alpha_{22}}{2}+\frac{\alpha_{23}}{3}+\frac{\alpha_{24}}{4}+\frac{\alpha_{31}}{1}+\frac{\alpha_{32}}{2}+\frac{\alpha_{33}}{3}+\frac{\alpha_{34}}{4}}{12}\\ \; & =40.14\;\mathrm{mm} \end{array}$$

(25) $$\begin{array}{cc} E_{l}=\bar{E_{l}} & =\frac{\frac{\alpha_{11}^{\prime }}{1}+\frac{\alpha_{12}^{\prime }}{2}+\frac{\alpha_{13}^{\prime }}{3}+\frac{\alpha_{14}^{\prime }}{4}+\frac{\alpha_{21}^{\prime }}{1}+\frac{\alpha_{22}^{\prime }}{2}+\frac{\alpha_{23}^{\prime }}{3}+\frac{\alpha_{24}^{\prime }}{4}+\frac{\alpha_{31}^{\prime }}{1}+\frac{\alpha_{32}^{\prime }}{2}+\frac{\alpha_{33}^{\prime }}{3}+\frac{\alpha_{34}^{\prime }}{4}}{12}\\ \; & =43.78\;\mathrm{mm} \end{array}$$

According to the data analysis in Table 3 and Table 4, the longer the robot travels forward, the greater the deviation from the straight line. TALBOT has less error in the track mode than in the leg mode. However, the positioning error ***E*** in both modes is less than 0.1 m, which meets the requirements of robot positioning and mapping.

### 4.2. Turning

**Figure 15:**
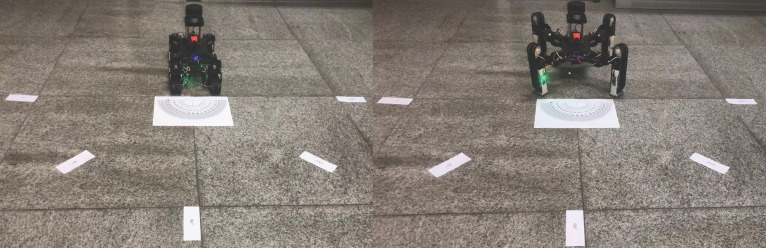
The turning experiment in tracked and legged modes.

We controlled robot turns $\frac{\pi }{4}$, $\frac{\pi }{2}$, $\frac{3\pi }{4}$, and π in place in tracked and legged modes; then, we recorded the error of the actual rotation angle and the odometer rotation angle. The turning experiment is shown in Figure 15. The experiments result are shown in Table 5 and Table 6.

The calculation formula for the control accuracy $\omega_{l}$, $\omega_{t}$ in the tracked mode and the legged mode is as follows:

(26) $$\begin{array}{cc} \omega_{t}=\bar{\omega_{t}} & =\frac{\frac{\varphi_{11}}{\pi /4}+\frac{\varphi_{21}}{\pi /2}+\frac{\varphi_{31}}{3\pi /4}+\frac{\varphi_{41}}{\pi }+\frac{\varphi_{12}}{\pi /4}+\frac{\varphi_{22}}{\pi /2}+\frac{\varphi_{32}}{3\pi /4}+\frac{\varphi_{42}}{\pi }+\frac{\varphi_{13}}{\pi /4}+\frac{\varphi_{23}}{\pi /2}+\frac{\varphi_{33}}{3\pi /4}+\frac{\varphi_{43}}{\pi }}{12}\\ \; & =-0.90^{{}^{\circ}}/\text{rad} \end{array}$$

(27) $$\begin{array}{cc} \omega_{l}=\bar{\omega_{l}} & =\frac{\frac{\varphi_{11}^{\prime }}{\pi /4}+\frac{\varphi_{21}^{\prime }}{\pi /2}+\frac{\varphi_{31}^{\prime }}{3\pi /4}+\frac{\varphi_{41}^{\prime }}{\pi }+\frac{\varphi_{12}^{\prime }}{\pi /4}+\frac{\varphi_{22}^{\prime }}{\pi /2}+\frac{\varphi_{32}^{\prime }}{3\pi /4}+\frac{\varphi_{42}^{\prime }}{\pi }+\frac{\varphi_{13}^{\prime }}{\pi /4}+\frac{\varphi_{23}^{\prime }}{\pi /2}+\frac{\varphi_{33}^{\prime }}{3\pi /4}+\frac{\varphi_{43}^{\prime }}{\pi }}{12}\\ \; & =-1.08^{{}^{\circ}}/\text{rad} \end{array}$$

During the movement of the robot, whether it is in the tracked mode or in the legged mode, TALBOT will be affected by friction and other system errors, which will affect the actual control effect. In the case of a given robot turning angle, testing the actual turning angle, TALBOT’s control accuracy in the tracked mode and the leg mode were both less than 2°/rad, which ensures the robot’s control effect in the turning situation.

### 4.3. Optimized Fast-Slam Evaluation

In order to verify the effect between the calibrated data of the odometer and the original data, the TALBOT was controlled to run in the indoor test site. We recorded the original odometer pose and the LiDAR estimated pose, and obtained the correction matrix *x*

(28) $$x=\left[\begin{array}{ccc} 0.965078 & 0.0296588 & -0.0014088\\ 0.0162602 & -0.677628 & -0.0377067\\ 0.0107093 & 11.8625 & 0.995829 \end{array}\right]$$

**Figure 16:**
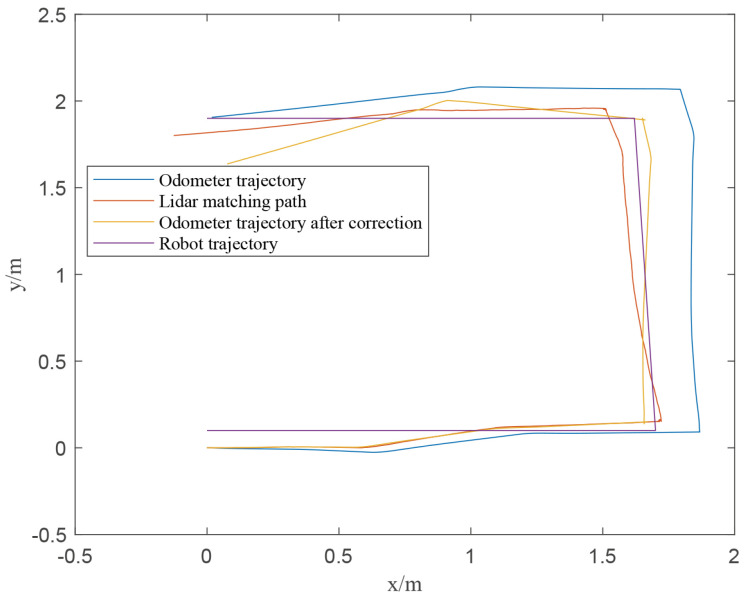
The corrected odometer position posture.

We multiplied the correction matrix *x* by the odometer data to obtain the corrected odometer position posture, as shown in Figure 16.

It can be seen from Figure 16 that as the distance increases, the difference between the odometer pose and the LiDAR pose becomes larger and larger. Since LiDAR has environmental observation information as the posterior distribution, the LiDAR odometer is more accurate than the wheel odometer. The corrected odometer positioning posture in the figure is closer to the LiDAR odometer posture, so after the odometer calibration, it can ensure that TALBOT has a higher positioning accuracy.

**Figure 17:**
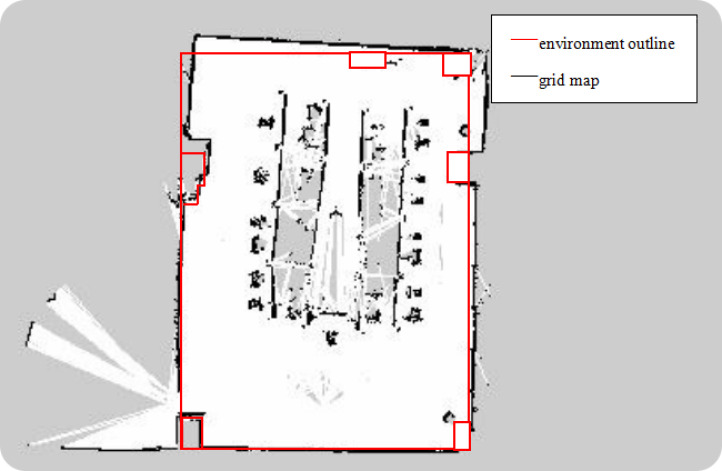
The grid map constructed by traditional Gmapping mapping algorithm in a small indoor environment.

**Figure 18:**
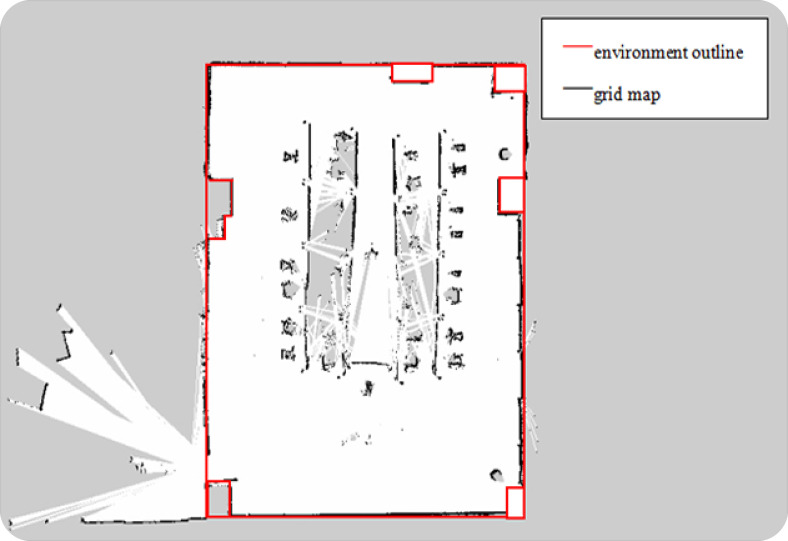
The grid map constructed by the optimized Gmapping mapping algorithm in a small indoor environment.

After fusing the odometer and IMU data, TALBOT in the tracked mode simulates the situation without GPS, using the traditional and the optimized Fast-Slam Gmapping algorithm to perform a comparative test of synchronous positioning and mapping. The mapping code ran on RaspberryPi; the operating system was Ubuntu 16.04; and the Rviz visualization plug-in was used to display the mapping effect. The effect of the raster map constructed by the traditional Gmapping mapping algorithm is shown in Figure 17, and the raster map constructed by the optimized Fast-Slam Gmapping algorithm is shown in Figure 18.

**Figure 19:**
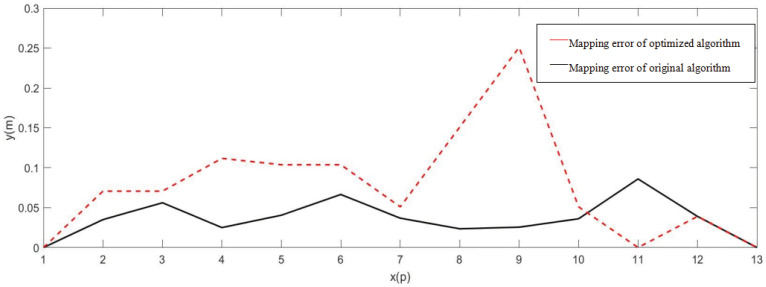
Error Analysis of Original Algorithm and Optimized Algorithm.

As can be seen from Figure 17, Figure 18 and Figure 19, we selected several contour points at the corresponding positions of the grid maps established by the two algorithms and took the distance from the grid map contour points to the corresponding points on the real environment contour as the mapping error. The grid map can basically describe the map of the test site, and the obstacle detection was accurate, but the average error of the original algorithm was about 10 cm, and the maximum error was 25 cm. The stability of the mapping accuracy was poor, and distortion and deformation appeared. The average error of the Gmapping algorithm after odometer calibration, integration of inertial components, and optimization was about 5 cm, and the maximum error was 8 cm. The mapping accuracy was relatively stable. Compared with the previous mapping, the boundaries were smoother; the abnormal points were reduced; and the straightness was better. However, there are still some shortcomings in mapping. Affected by the height of the LiDAR, only obstacles at the same height can be detected. Too-fast speed will cause problems such as map distortion, deformation, and breakage.

## 5. Conclusions

This article reports a Track-Leg Transformable Robot (TALBOT). The robot adapts to different terrains by changing the motion mechanism. When the ground is relatively flat, the tracked mode has higher efficiency, and when the ground is rugged, the robot can cross obstacles in the legged mode.

In addition, we conducted research on the motion control of the robot. When the robot is in the tracked mode, the robot drives the tracks to move. By adjusting the speed of the tracks on two sides, the robot is controlled to complete the movement in different directions. When the robot is in the legged mode, the hopf vibrator is used as the central pattern generator of the tracked leg. The hopf vibrator acts on the hip and knee. The ankle joints of the tracked leg is controlled by the mapping of the knee joint. After the four vibrators are coordinated with each other, the gait of the robot can be changed by changing the parameters of the vibrator. This article mainly discusses the walk and trot gaits. By adjusting the frequency $\omega_{sw}$ of the swing phase, the forward speed of the robot is adjusted. The gait is converted by adjusting the support factor β. By adjusting the amplitude of hopf, the robot can be steered.

The experiments show that the robot has good control accuracy in straight and turning motions. In the tracked mode, the robot moves straight for 4 m and deviates from the straight line by only 183 mm. In the in-situ turning motion, the robot’s control accuracy was −0.90°/rad, which meets the robot’s control requirements. When the robot is in the legged mode, the robot moves straight for 4 m and deviates from the straight line by 183 mm. In the turning movement, the robot’s control accuracy was −0.90°/rad.

In addition, the robot is also equipped with LiDAR. The robot is equipped with LiDAR, which can build a map of the surrounding environment. We used the least square method to calibrate the odometer and fuse the wheel odometer and IMU to preprocess the sensor data, which obtain more accurate pose information than a single wheel odometer and reduce the non-systematic error in relative positioning. Then, we used the coordinate transformation relationship of the robot, and the environmental information obtained by the external sensor and the motion information of the robot were transformed into the world coordinate frame. The proposal distribution was optimized based on the particle filter algorithm. LiDAR matching represented the proposal distribution, and the sampling range was limited to a relatively small range, which improves the mapping effect. By comparing the mapping effects before and after optimization, the experiments showed that the optimized algorithm has smooth mapping boundaries, fewer abnormal points, and better straightness.

TALBOT is an experimental prototype. Due to its size, the robot’s obstacle-climbing performance is limited. A larger size robot is currently being developed to make the robot better able to adapt to the terrain. In addition, related studies on the control of brushless motors are also underway to replace the current servos.

## Figures and Tables

**Table 1:** The rotation angle of servo.

Steering Gear	The Angle
Hip joint servo	$\frac{\pi }{3}$∼$-\frac{\pi }{3}$
Knee joint servo	$\frac{\pi }{3}$∼$-\frac{\pi }{3}$
Ankle joint servo	$\frac{\pi }{6}$∼$-\frac{5\pi }{6}$

**Table 2:** Robot specifications.

Length	Tracked mode	448 mm
	Legged mode	428 mm
Width	Tracked mode	213 mm
	Legged mode	382 mm
Height	Tracked mode	288 mm
	Legged mode	305 m
Total weight		5.15 kg
Tracked foot structure	Length	212 mm
	Width	58 mm
	Height	46 mm
Sensor	Encoder	(×4) Giant Magneto Resistance sensor
	LiDAR	RPLIDAR-A1
Processer	Raspberry Pi	B3
	Stm32	f103
Actuator	Driving	(×4) 4 W DC motor
	Steering mechanism	(×12) 5 W digital Servo
		The minimum angle is 0.2823 degrees
Battery	12.6 V Li-ion battery	20 min continuous run time

**Table 3:** Results of straight line motion experiment in tracked mode.

Distance (m)	Deviation Distance α (mm)			Deviation Angle (°)		
	1st Set	2nd Set	3rd Set	1st Set	2nd Set	3rd Set
1	25	27	31	0.79	0.92	0.88
2	83	88	90	1.13	1.20	1.23
3	125	132	130	1.34	1.41	1.38
4	185	189	183	1.58	1.56	1.60

**Table 4:** Results of straight line motion experiment in legged mode.

Distance (m)	Deviation Distance $\alpha^{\prime }$(mm)			Deviation Angle (°)		
	1st Set	2nd Set	3rd Set	1st Set	2nd Set	3rd Set
1	27	32	29	0.95	1.02	0.98
2	94	98	97	1.39	1.52	1.42
3	142	148	150	1.59	1.60	1.57
4	192	198	195	1.85	1.86	1.82

**Table 5:** Results of turning experiment in tracked mode.

Angle/(rad)	Deviation Angle φ (°)		
	1st Set	2nd Set	3rd Set
$\frac{\pi }{4}$	−0.5	−0.7	−0.7
$\frac{\pi }{2}$	−1.6	−1.5	−1.6
$\frac{3\pi }{4}$	−2.0	−2.1	−2.3
π	−2.8	−2.7	−3.0

**Table 6:** Results of turning experiment in legged mode.

Angle/(rad)	Deviation Angle $\varphi^{\prime }$ (°)		
	1st Set	2nd Set	3rd Set
$\frac{\pi }{4}$	−0.7	−0.9	−1.0
$\frac{\pi }{2}$	−1.9	−2.1	−2.0
$\frac{3\pi }{4}$	−2.3	−2.3	−2.5
π	−2.9	−2.8	−3.0

## Data Availability

Data sharing not applicable.

## Abbreviations

The following abbreviations are used in this manuscript:

MDPI	Multidisciplinary Digital Publishing Institute
DOAJ	Directory of Open Access Journals
CPG	Central Pattern Generator
SLAM	Simultaneous Localization and Mapping
IMU	Inertial Measurement Unit
EKF	Extended Kalman Filter
